# Scalable Steady State Analysis of Boolean Biological Regulatory Networks

**DOI:** 10.1371/journal.pone.0007992

**Published:** 2009-12-01

**Authors:** Ferhat Ay, Fei Xu, Tamer Kahveci

**Affiliations:** Computer and Information Science and Engineering, University of Florida, Gainesville, Florida, United States of America; Center for Genomic Regulation, Spain

## Abstract

**Background:**

Computing the long term behavior of regulatory and signaling networks is critical in understanding how biological functions take place in organisms. Steady states of these networks determine the activity levels of individual entities in the long run. Identifying all the steady states of these networks is difficult due to the state space explosion problem.

**Methodology:**

In this paper, we propose a method for identifying all the steady states of Boolean regulatory and signaling networks accurately and efficiently. We build a mathematical model that allows pruning a large portion of the state space quickly without causing any false dismissals. For the remaining state space, which is typically very small compared to the whole state space, we develop a randomized traversal method that extracts the steady states. We estimate the number of steady states, and the expected behavior of individual genes and gene pairs in steady states in an online fashion. Also, we formulate a stopping criterion that terminates the traversal as soon as user supplied percentage of the results are returned with high confidence.

**Conclusions:**

This method identifies the observed steady states of boolean biological networks computationally. Our algorithm successfully reported the G1 phases of both budding and fission yeast cell cycles. Besides, the experiments suggest that this method is useful in identifying co-expressed genes as well. By analyzing the steady state profile of Hedgehog network, we were able to find the highly co-expressed gene pair GL1-SMO together with other such pairs.

**Availability:**

Source code of this work is available at http://bioinformatics.cise.ufl.edu/palSteady.html twocolumnfalse]

## Introduction

Analyzing biological networks is essential in understanding the machinery of living organisms which has been a main goal for scientists [1], [2]. Gene regulatory networks and signaling pathways are two important network types that play role in every process of living organisms [3]. In the last decade, significant amount of research has been done on reconstruction of these networks from experimental data [4]–[11]. The amount of regulatory data produced by these methods is sufficient enough to trigger the research on automated tools to analyze various aspects of these networks. We use the term *biological regulatory networks (BRN)* to combine gene regulatory networks and signal transduction pathways.

To capture the biological meaning of BRNs, it is necessary to characterize their long term behavior. A common way to achieve this is to identify the *steady states* of the dynamic system defined by a BRN. Identification of steady states of BRNs is crucial in several applications such as the treatment of various human cancers [12], [13] (e.g. leukemia, glioblastoma) and genetic engineering [14]. Additionally, the steady state analysis has proven to be successful to explain the flower morphogenesis of *Arabidopsis thaliana* [15]–[17], the differentiation process of T-helper cells [18]–[20], the mechanism of T cell receptor signaling [21] and the cell cycles of yeast types [22], [23].

We use Boolean values for the states of the genes (“ON” or “OFF” meaning high or low activity) since it is successfully used in the literature for BRNs [15], [18], [20], [22], [23]. Recently, several methods have used categorical values (e.g., low, medium, high activity) for gene states in their model [16], [24], [25]. The steady states extracted by these methods showed high parallelism with the ones found using Boolean models. The naive approach to steady state identification in Boolean networks is to exhaustively search the state space. However, the number of possible states of a BRN is exponential in the number of its genes. Therefore, exhaustive methods are computationally infeasible for even moderately sized BRNs. To address this problem, some existing methods use finite-state Markov chains [26], binary decision diagrams (BDD) [18], [19], constraint programming [27], probabilistic Boolean networks [28], linear programming [29], relational programming [30] and module networks [31], [32].

Orthogonal to the selection of the computational method, there are two commonly used alternatives for modeling the state transitions. These are *synchronous* and *asynchronous* models and both are used in the literature [18], [19], [27], [30]. Synchronous models assume that the activity levels of all the genes change simultaneously. Hence, the next state is deterministically decided by the current state. On the other hand, asynchronous models consider time in small intervals, such that only one gene can change its state at an interval and state change is equally likely for all genes [19]. For an 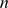 gene BRN, the state space of synchronous model has 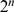 states and 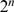 state transitions. For asynchronous model, the number of states is still 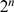 but the number of possible transitions can go up to 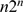. The advantages/disadvantages of these models together with their effect on running time of steady state identification algorithms are discussed in the literature [19], [33], [34]. Due to its strong assumptions, such as all genes change their state at the same time and all have equal response times to these changes, synchronous model is arguably more of an abstraction of the biological process compared to asynchronous model. We use the asynchronous model in our discussion here, however, it is important to note our method works for the synchronous model as well.

A *state of a BRN* is the union of the states of its genes at a certain time. The state of a gene can change over the time due to internal regulations or external stimulants. *Steady states* are the states in which the dynamic system of that BRN stabilizes. The rest of the states of the network are called *transient states* and they are usually not of interest from biological viewpoint. *We follow the steady state definition of Garg* et al.[*18*].

### Definition 1

*Let* 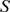 *be a set of states. Each* 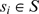 *is steady if and only if the following conditions are satisfied:*

- *The set of the successor states of all the states in* 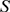 *is equal to* 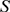

- *For each* 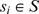 *once it is visited the probability of revisiting* 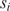 *is equal to 1 in a finite number of state transitions.*

**Figure 1:**
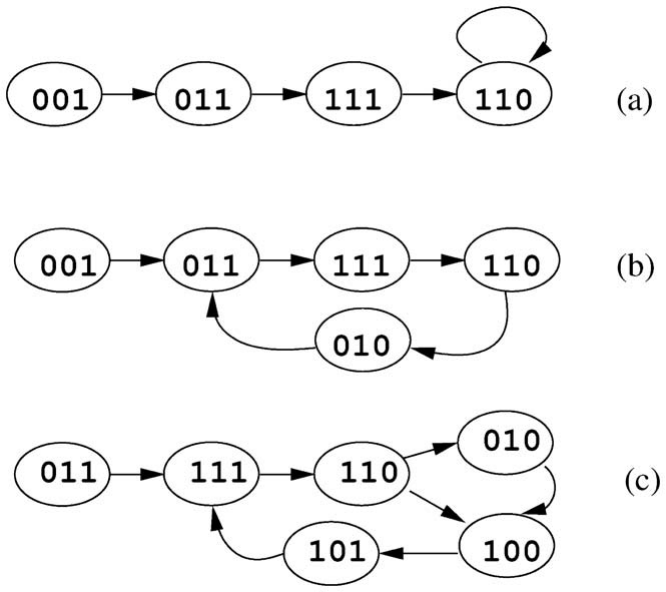
States of a hypothetical network with three genes. The binary values correspond to activation levels of these genes. (a) The three states on the left are transient and of Type 1. The state with self loop is steady and Type 0. (b) The four states in simple loop are cyclic steady states and they are of Type 1. (c) The leftmost state is transient and Type 1. Even though only 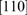 is of Type 2 (others are Type 1), the remaining five states create a complex loop, and thus they are transient.

This definition suggests that there are two types of possible steady states, self loops (e.g., Figure 1(a)) and simple loops (e.g., Figure 1(b)) as named in [18]. If a set of states create a complex loop, then all the states of this set are transient since at least one of the states does not satisfy the second condition of the above definition. For instance, in Figure 1(c) the state 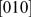 is not revisited with probability equal to 1 in finite steps since the system can loop forever through other four states which create a loop. Similarly, Garg *et al.* name such sets of states as transient states. Figure 1 exemplifies all the state types discussed above.

### Our Contributions

In this paper, we develop an algorithm that identifies all the steady states of BRNs accurately and efficiently.

To mathematically express this problems clearly, we define three types of states according to the number of possible outgoing transitions from them. We name a state *Type 0* if it has no outgoing transitions to another state except itself (self loop) (state 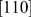 in Figure 1(a)). A state with exactly one outgoing transition to another state is *Type 1* (all the states in Figure 1(b)). States with more than one outgoing transitions are *Type 2* states (state [110] in Figure 1(c)). Using this notation, we observed the following:

- All Type 0 states are steady (self loops).
- All Type 2 states are transient.
- All the states of a simple loop are of Type 1.

It is important to note that all the above observations are one-sided (i.e. “if” conditions). For instance, second observation means that if a state is of Type 2 then it is transient. However, a transient state does not have to be a Type 2 state. Here, we name the steady states of Type 1 as *cyclic steady states* (i.e, simple loops). Our method first divides the whole state space into three types (Type 0, 1 and 2) without materializing the exponential state space graph. Then, we extract the cyclic steady states from Type 1 states by using a randomized traversal method. Cyclic steady states together with the Type 0 states constitute all the steady states of the BRN of consideration.

We use the Boolean network model proposed by Kauffman *et al.* [35]. We build a hypothetical state transition graph using the interactions in a BRN. We develop a mathematical model that uses binary decision diagram (BDD) data structure [36] to classify each state into one of the three classes, namely Type 0, Type 1 and Type 2. Type 0 and Type 2 states are guaranteed to be steady and transient (i.e. not steady), respectively. Type 1 states can be either one. To further classify the Type 1 states as transient or steady, we develop a randomized traversal method which samples random seed states from Type 1 states and classifies the visited states during the traversal from this seed state. While sampling, we calculate the estimators for the number of steady states, expected steady state distribution of individual genes and joint-steady state distributions of gene pairs. We calculate a stopping criterion from the statistical information of explored states. This criterion allows early termination of sampling when the user defined percentage of steady states are found with high confidence. In summary, our technical contributions are:

- We build a mathematical model for pruning a very large portion of state space quickly without losing any steady states.
- We develop a randomized traversal method that computes estimators for the number of steady states and the fraction of individual genes and gene pairs being active in these states in an online fashion. Our algorithm guarantees to find all the steady states after sufficient number of iterations.
- We formulate a stopping criterion which uses the information of classified states to terminate the algorithm when sufficient percentage of steady states are extracted with a given confidence value.

## Results and Discussion

### Cell Cycles of Budding Yeast and Fission Yeast

To evaluate the accuracy of the results reported by our algorithm, we compared the steady states that we found to the steady states that are reported in the literature. For this purpose, we use the cell cycle networks of two yeast types, namely *Saccharomyces cerevisiae* (budding yeast) and *Schizosaccharomyces pombe* (fission yeast). We consider the key regulatory genes of these networks since the core process of these two cell cycles are well analyzed in the literature by both differential equation models [37], [38] and Boolean network models [22], [23], [39], [40].

The cell cycles of both yeasts go through four main phases. In the first phase the yeast cell grows till its size reaches a certain amount (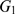). The second phase is when the DNA is synthesized and chromosomes are replicated (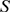). Third phase is a transition gap between the second and fourth (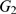). The cell division is completed at the fourth phase named 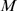. The two new cells then enter the 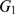 phase again which completes the cycle. The state corresponding to 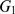 phase is a steady state that is observed the most in the yeast life cycle.

**Figure 2:**
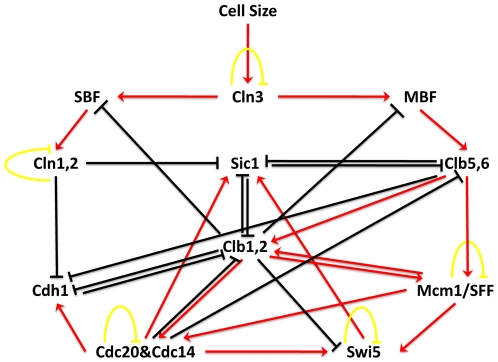
Regulatory network of the cell cycle of budding yeast. Red arrows with pointed heads represent activation, black arrows with bar heads represent inhibition and yellow arrows indicate self-degradation.

**Figure 3:**
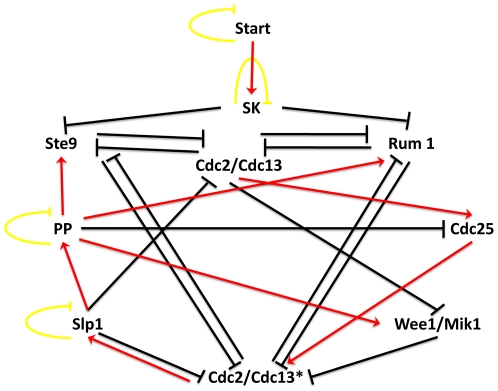
Regulatory network of the cell cycle of fission yeast. Red arrows with pointed heads represent activation, black arrows with bar heads represent inhibition and yellow arrows indicate self-degradation.

Li *et al.* [23] studied the Boolean network model of the budding yeast (Figure 2) and identified the Boolean states visited during a complete cell cycle together with seven steady states of the network corresponding to the fixed points of the dynamic system. Similarly, Davidich *et al.* [22] found thirteen different steady states for the Boolean model of the cell cycle of fission yeast (Figure 3).

Here we compare the steady states reported by our method with the ones from the methods of Li *et al.* and Davidich *et al*. For this we use vector notation to represent the activity levels of an ordered gene set. In this notation, 0 means the corresponding gene is inactive, 1 means its active and X means it can be either one. For instance, for a gene set of 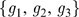, the [01X] vector represents two states, namely [010] and [011].

The budding yeast cell cycle network in Figure 2 is the same as the one analyzed by Li *et al.* [23]. We use the order {*Cln3, MBF, SBF, Cln1-2, Cdh1, Swi5, Cdc20, Clb5-6, Sic1, Clb1-2, Mcm1*} for the vector representation of the states of eleven genes in this network. We follow Li *et al.* by excluding *Cell size* from the gene set and the state representation. Li *et al.* reported seven steady states for this network one of which corresponds to the 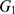 phase of the cell cycle. We identified eight different steady states, six of which are Type 0 and the other two are of Type 1. Six Type 0 steady states we found are [0000X000X00] (4 states) and [0100X000100] (2 states) and all are also reported by Li *et al*. Also, our method accurately labeled the [00001000100] state that corresponds to 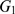 phase as steady. The two Type 1 steady states which visit each other in a cycle are 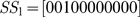 and 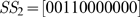. 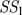 is when *SBF* is the only active gene in the network. 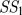 is followed by 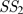 since in the next time step *SBF* also activates *Cln1-2*. Due to self degradation of *Cln1-2* in state 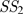, this state goes back to the 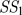 again. The method of Li *et al.* labels 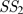 as steady whereas it does not report 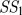.

For the states of the fission yeast cell cycle in Figure 3, we use the ordered gene set {Start, SK, Cdc2/Cdc13, Ste9, Rum1, Slp1, Cdc2/Cdc13*, Wee1/Mik1, Cdc25, PP}. Our method reports fifteen different steady states all are of Type 0. These states are: [0001X00XX0] (8 states), [0000100XX0] (4 states), [00000001X0] (2 states) and [0000000000]. The first set of states contains the steady state [0001100100] that corresponds to most stable phase (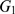) of the cell cycle. This state together with twelve other steady states we found matches exactly the ones found by Davidich *et al.* [22] The two additional steady states that we found different than Davidich *et al.* are [0000000100] and [0000000110]. The first state corresponds to high activation level of only Wee1/Mik1 genes and the second state is when Cdc25 is also active together with Wee1/Mik1. The reason of this difference is that Davidich *et al.* manually sets a negative threshold for Cdc2/Cdc13 activation. Cdc2/Cdc13 degrades Wee1/Mik1 which prevents their system from visiting the two steady states we found without setting any threshold manually.

*These two examples suggest that our method can accurately identify the steady states of BRNs.*

### Performance Evaluation

Here, we compare the performance of our method to that of Garg *et al.* [18], [19]. We used the asynchronous state transition model for both algorithms in this experiment. We compared the running times for a number of real BRNs as well as for randomly generated networks. We compiled the real BRNs from the pathway database PID [41] and other published work [18], [19], [22], [23]. Table 1 reports the running times for Garg *et al.*'s method named *Genysis* and our algorithm with different parameter settings.

**Table 1:** The comparison of our algorithm with an existing method, Genysis [18], [19],on real and random networks.

Network Name	Genes	Interactions	Genysis^1^	Our Algo.^2^	Our Algo.^3^
Fission yeast cell cycle [22]	10	27	0.21 s	0.18 s	0.17 s
Budding yeast cell cycle [23]	12	35	0.13 s	0.25 s	0.22 s
T-Helper cells [18]	23	35	0.23 s	1.14 s	0.43 s
p38 MAPK signaling [41]	26	28	545.3 m	11.3 s	2.1 s
T-cell receptor [19]	40	58	20.7 m	14.2 s	2.11 s
randomNet 1	20	32	10.4 m	2.282 s	0.3 s
randomNet 2	30	48	-	5.923 s	3.13 s
randomNet 3	40	64	-	4.7 m	3.4 m
randomNet 4	50	80	-	68.7 m	15.3 m

1 We used a cut-off time of 24-hours and “-” indicates that the method could not find all steady states within this time. 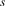 denotes seconds and 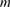 denotes minutes.

2 Running time of our algorithm when 90% of the steady states are found with 90% confidence.

3 Running time of our algorithm when 80% of the steady states are found with 80% confidence.

For real networks of small size such as yeast cell cycles and T-Helper network, the running times for both methods are around one second with Genysis running slightly faster than our method. However, for bigger real networks our method's running time is significantly smaller than Genysis. As the authors also stated in their work, Genysis might need extensive amount of running time when using asynchronous model due to their heuristics to select seed states from the state space. The row corresponding to *p38 MAPK signaling pathway* constitutes a good example for this scenario. For the same network our algorithm can identify the 90% of the steady states with 90% confidence in only 11.3 seconds. Additionally, the running times on four randomly generated networks indicated that Genysis can not scale well with the growing network size whereas our algorithm can still find large portion of the steady states in a few minutes. It is worthwhile to note that both Genysis and our algorithm have exponential time and space complexity in the worst case scenario. This is a direct consequence of using BDD data structure as it has exponential worst case complexity.

We also compared the steady states found by both algorithms for the two yeast cell cycles. As discussed in previous section, the steady states of these two networks are reported in Li *et al.* [23] and Davidich *et al.* [22]. For the budding yeast cell cycle in Figure 2, Genysis was able to identify only the trivial steady state when all the genes are inactive. For the fission yeast, Genysis labeled the state that corresponds to the 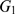 phase (only the genes *Ste9*, *Rum1* and *Wee1/Mik1* are active) of cell cycle as transient. As reported in Davidich *et al.* [22], 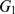 is the most stable phase of this cycle and our method correctly classifies this state as steady.

*The above results support that our algorithm is more scalable and practical compared to Genysis. Furthermore, the steady states we reported for yeast cell cycles match better with the previous findings.*

### Co-Expressed Gene Pairs in Human Hedgehog Network

We calculate the fraction of steady states in which two genes are in active state together. Biologically this fraction corresponds to the co-expression of the two genes. Revealing co-expressed genes has great significance in discovery of conserved genetic modules [31], [42], [43] and identification of differentially expressed genes [44].

Here, we compare the co-expression values for gene pairs found by our algorithm with the values reported in the gene co-expression database, COXPRESdb [45]. For this purpose, we use *The Hedgehog signaling network* of *Homo Sapiens* given in the KEGG Pathway Database [46]. This network consists of 17 genes and hence, 136 possible gene pairs. We sorted the gene pairs according to their co-expression values in decreasing order and compared our ordering with the one in COXPRESdb. We picked the top 20 gene pairs from our list and searched for the indices of these pairs in the ordering of COXPRESdb. Here, we report the largest index, 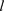, among these 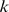 indices for different values of 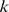.

For 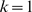 we have 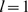, which means that the highest co-expressed gene pair (*GL1-SMO*) in our ordering is also the top scoring pair in COXPRESdb. For 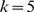 we have 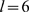, meaning that the five gene pairs (*GL1-SMO*, *GSK3B-FBXW11*, *RAB23-GAS1*, *GLI1-IHH* and *SUFU-SMO*) with the highest ranks in our ordering are in between the top 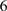 pairs in the ranking of COXPRESdb. For the other values of 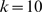 and 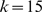, the 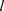 values are 16 and 35 respectively. Hence, the gene pairs reported by our method that are found to be active together in the steady states suggest that there is a co-expression between these two genes.

*The above results suggest that our algorithm is useful in predicting co-expression of genes by utilizing the the steady state information of BRNs.*

### Accuracy of Estimators

To evaluate the quality of our sampling-based estimators, we measured their correctness and convergence rate. Correctness means that the estimates will eventually converge to the correct value. For the convergence rate, a good estimator should approximate the correct value after a small fraction of the state space is explored.

**Figure 4:**
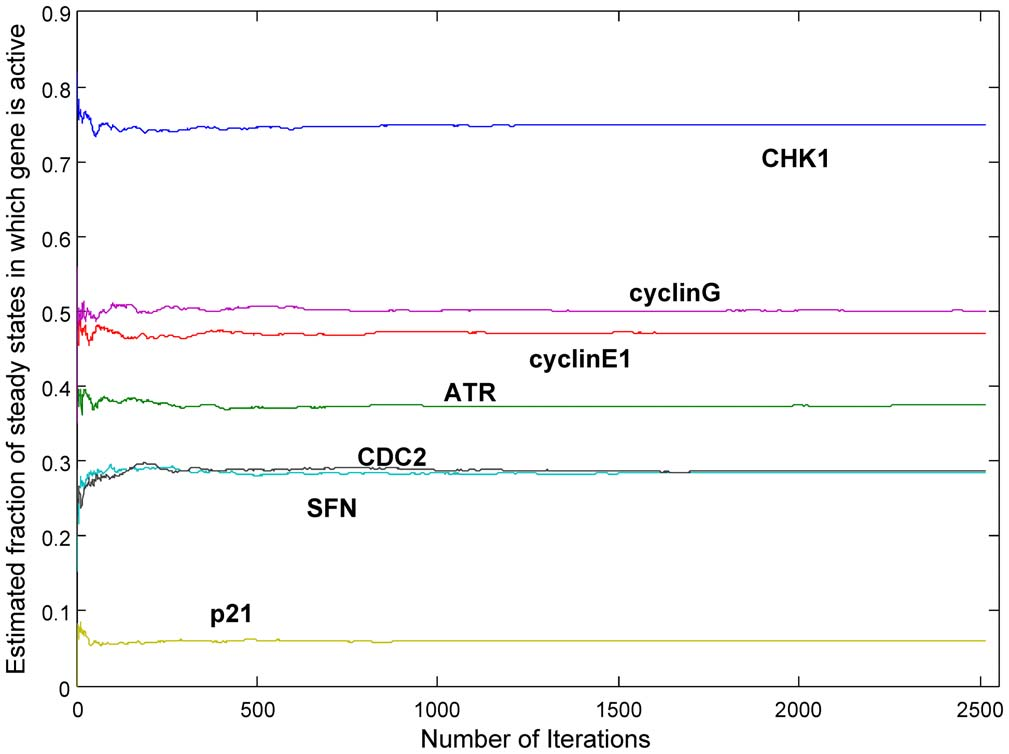
Convergence of the estimators for the steady state profiles of the genes. These genes are a selected subset of the genes of *p53 network* of *Homo Sapiens* [46]. Y-axis shows for each gene the fraction of steady states that the gene is in active state.

We use a portion of *p53 network* of *Homo Sapiens* taken from KEGG [46] in this experiment. We measure the estimated number of steady states at which a gene is active for each gene at each iteration of our algorithm. Our algorithm traverses the entire space of Type 1 states in about 2,500 iterations for this network. Figure 4 shows the results for seven different genes. We plot these genes as they have different steady state profiles. In other words, they vary in the fraction of steady states in which they are active (e.g. CHK1 is active whereas p21 is suppressed in most of the steady states). The results show that our estimators converge to the correct ratio for all genes in less than 500 iterations. The rapid convergence suggests that our algorithm approximates the correct profile of gene levels at steady states without traversing the whole space of Type 1 states. This suggests that, equipped with the stopping criterion we devised, our algorithm is also practical and accurate for BRNs with large number of Type 1 states since early termination of the algorithm does not lead to significant deviation from the correct steady state profile.

## Methods

This section discusses our algorithm for identifying all the steady states of Boolean BRNs. First we describe the mathematical model for expressing the states and state transitions. Then, we discuss our method to segregate the state space into three subspaces. Finally, we present our randomized traversal method that extracts Type 1 steady states. We also give the formulation of a stopping criterion that terminates the traversal when sufficient amount of steady states are reported with high confidence.

### State Transition Model

In order to identify the steady states of a BRN, we first need to build a mathematical model that explains its states and how the network moves from one state to another.

Let 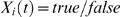 denote the state of the 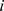
th gene at time 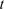. Here *true* denotes that 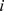
th gene is “active” and *false* denotes that it is “inactive”. We use 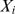 instead of 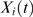 for simplicity wherever appropriate.

We summarize the interactions that determine the next state of the 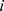
th gene from the activity values at time 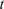 as follows. The 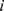
th gene will be inactive if at least one of its suppressors is active. If all the suppressors of the 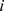
th gene are inactive and at least one of its activators is active, then it becomes active in the next time step. In all other situations the state of the 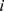
th gene remains unchanged. Even though the assumption that one inhibitor can suppress all activators seems questionable, it is commonly observed in biological networks. Wu *et al.* [40] named this as “strong inhibition” model and showed that it produces the same results as threshold network model [39] for fission yeast cell cycle network. Also, it has been used as a modeling decision by Garg *et al.* [18], [19]. However, it is important to note that our method does not depend on this assumption.

The following equation summarizes how the next state of 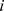
th gene is determined:
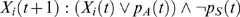
(1)


In this equation, the symbols 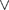 and 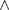 denote the logical “OR” and “AND” operators, 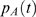 and 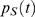 represent predicates for the activators and the suppressors of the 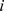
th gene at time 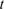, respectively. We compute these predicates as 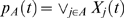 and 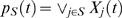, where 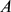 and 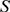 are the sets of indices for activators and the suppressors of the 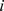
th gene.

An important observation is that, even though the next state of the 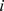 th gene is deterministically calculated, there can be multiple next states for the whole network since we use asynchronous model. A state of a given BRN is defined by the states of individual genes. Let 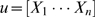 denote a state of the network. The network can move from state 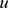 to state 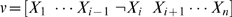 only if the 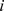
th gene is one of the genes that can have a state change. Individual genes that can issue a state change at a given state determines the possible next states of the network.

We model the changes in the states of a BRN using an abstract graph representation. In this graph, each vertex corresponds to a possible state of the BRN. Thus, if there are 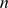 genes in a BRN, then the corresponding graph contains 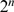 vertices. There is an edge from vertex 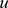 to vertex 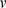, if it is possible to change the state of the BRN from the state represented by 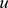 to the state represented by 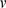 by only changing the state of a single gene. There can be up to 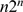 edges between these states. This graph is hypothetical as we use it only for building our mathematical model. We never materialize this exponential graph in our method.

We classify the vertices of this graph into three classes based on the number of their outgoing edges. Figure 1 provides visual examples for all three state types listed below:

- Type 0: The vertices that have no outgoing edges (except self cycles). These vertices correspond to steady states as the state of the network cannot change once one of them is visited. (Figure 1(a))
- Type 1: The vertices that have exactly one outgoing edge. The states for these vertices can be steady or transient. (Figure 1(b))
- Type 2: The vertices that have two or more outgoing edges. All Type 2 states are transient. (Figure 1(c))

In the following section, we describe our method for segregating the state space into the above three types.

### Segregation of States using BDDs

As we discussed in the previous section, we never generate the state transition graph of the input network. A simple observation on our state transition model allows us to segregate the states without this materialization. This segregation results in not only the immediate identification of all Type 0 steady states, but also eliminates a huge portion of states by classifying them as transient.

For instance, for *T-Helper cell network* with 23 genes and 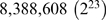 possible states, our segregation method classifies 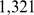 states as Type 0 and 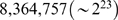 states as Type 2 in only 0.08 seconds. The remaining 22,530 states are labeled as Type 1. Thus, we need to explore only a small percentage (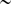
0.26%) of the whole state space.

Here, we describe how we construct the BDDs for all Type 0 states and all Type 1 states, namely 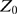 and 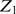. We first define a predicate that will be handy in this discussion.
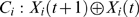
(2)


Here, 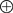 denotes the logical “XOR” operator. 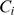 evaluating to true at time 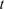 means that gene 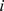 will change its state from 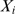 to 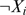 at time 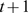. Otherwise, it preserves its current state. The following equations, show the formulas of BDDs representing Type 0 and Type 1 states:
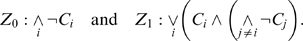




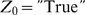
 represents the states that do not satisfy any of the 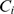 conditions (i.e. none of the genes change state). The states in 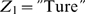 satisfy exactly one of the 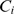 conditions (i.e. exactly one gene changes state). The states which are not included in the two BDDs above are called Type 2 and they are all transient states. The BDD for these states can be constructed similarly. However, we simply eliminate these states since they do not reflect the long term behavior of the system. By doing this without materialization, we quickly reduce the state space of the problem to a significantly smaller one. In the next section, we describe how we extract the steady states of Type 1.

### Extracting Cyclic Steady States

In this section, we develop a randomized traversal strategy that identifies the steady states of Type 1. We call these states “cyclic steady”. An example for this is the cycle of four states in Figure 1(b). At the end of each traversal, we remove the traversed states from the state space that by using difference operator of BDD. In other words, our method avoids redundant enumeration of the states. After traversing a portion of the vertices, we estimate the total number of steady states, the probability of each gene being active and the joint probability of gene pairs being co-expressed in steady states. It is worth mentioning that our traversal method never traverses a state more than once. Hence, if it runs for enough time it labels all the Type 1 states as steady or transient. Algorithm 1 briefly describes how we traverse the Type 1 states. Next, we elaborate on different steps of this algorithm.

### Step 1. Selecting a random seed state

We obtain a random seed state among the untraversed satisfying assignments of the BDD for Type 1 states. We do this by traversing the BDD from root node to the leaf level. At each step of the traversal, we randomly pick a child node of the currently visited node. When we reach the leaf level of the BDD, the states of all the genes are determined and hence, our seed state for the whole BRN.

**Algorithm 1** Randomized traversal of Type 1 states

1. Randomly get an unobserved vertex from the Type 1 set.
2. Follow the outgoing edge to traverse the graph until seeing one of the following vertices
  - A vertex that is labeled as transient or steady in previous iterations.
  - A vertex that is traversed in this iteration.
3. Label all the traversed vertices as transient or steady and update the estimators.
4. Stop if the number of steady states observed so far is *sufficient*.

### Step 2. Traversal starting from the seed state

Once we choose an unobserved seed state, the next step is to understand whether or not we can reach to a new steady state from this state. To do this, we traverse the state transition graph starting from this vertex by following the edges.

**Figure 5:**
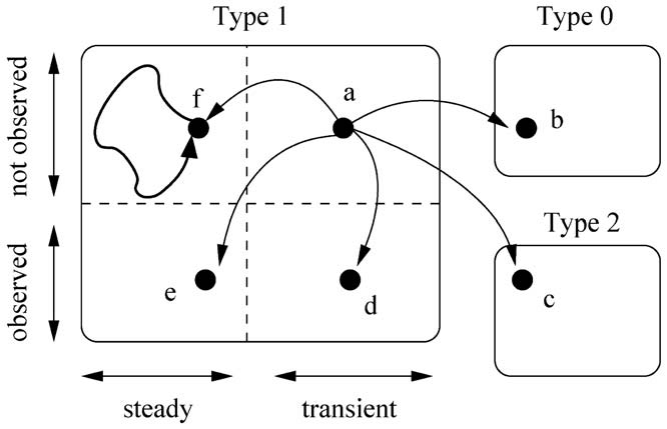
Summary of the traversal process for a randomly picked state 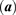 from unobserved Type 1 states. If the path starting from a ends at 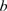, 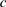, 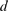 or 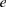, then all the states on this path are transient (Step 2(i) of Algorithm 1). If the path starting from 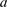 ends at a state like 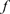 then all the states on the path from 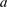 to 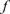 are transient (excluding 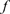) and all the states on the cycle from 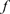 to 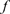 are steady.

Since the seed state is of Type 1, by definition, it has only one outgoing edge. Thus, we can easily find the next state as the state that satisfies the transition condition. We continue traversal by applying the same principle. Figure 5 summarizes the possible cases that can occur during this traversal. Starting from an unobserved state if we traverse one of the following three paths then all the states visited on this path are transient:

- A path ending in a Type 0 state
- A path ending in a Type 2 state
- A path ending in a state that is observed in previous iterations

Notice that all three cases correspond to Step 2(i) of our traversal method. The next case produces both cyclic steady and transient states:

- A path leading to a cycle of states visited in current iteration

In this case, we label all the states on the cycle as steady and the other states on the path as transient. For instance, if the traversal starts from the [001] state in Figure 1(b), then [001] is transient and other four states are Type 1 steady states.

### Step 3. Calculating Estimators

At each iteration, we traverse a path in the state transition graph and label each state on this path as transient or steady. We name the set of vertices visited in each such traversal as an observation. Using these observations, we develop estimators for the total number and the “ *profile*” of steady states. The profile of the steady states is the vector where the 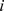
th entry is the expected fraction of the steady states at which the 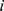
th gene is active. For example, if the second entry of the profile is 0.95, it means that we expect that the second gene is active in 95% of the steady states. We also compute the estimators for the joint expression (co-expression) fractions of gene pairs. Computing these estimates is important as they can lead to early prediction of the steady state profile.

Here, we describe in detail the calculation and the analysis of the estimator of the total number of Type 1 steady states. First of all, we prove that it is an unbiased estimator. Then, we discuss how to minimize the variance of this estimator. For the other estimators we only give the formulations.

First, let us introduce some notation we use throughout this section:

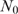
, 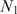: Number of Type 0 and Type 1 states, respectively. We calculate these numbers at the initial segregation step.

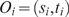
: 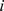 th observation. 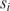 and 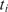 are the number of observed steady and transient states traversed in this observation.

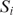
, 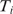, 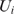: Total number of observed steady states, observed transient states and unobserved states after first 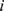 observations, respectively.

From the definitions above, we can calculate 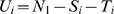, 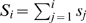 and 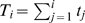. Now, we introduce a 0/1 random variable 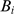 for each observation 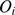. At a given time 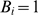 means the current iteration results in observation 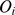. We simulate our sampling by assuming at any time one and only one of the 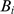
's can be 1. In other words, 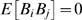 for any 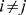. Notice that 
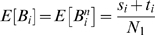
 for observation 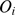. We formulate the estimator of the total number of Type 1 steady states at the 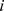 th iteration as:
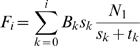
(3)


#### Lemma 1

*The estimator* 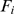 *is an unbiased estimator.*

#### Proof

We prove this by showing the expected value of 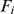 is equal to the total number of Type 1 steady states. Taking expectations of both sides and replacing 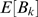 with 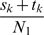:
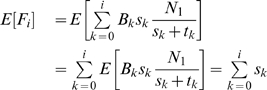



After defining the estimator, the next step is to calculate its variance.

#### Lemma 2

*The variance of* 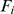 *is*

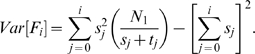



#### Proof

We know that, 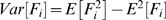. We first compute 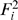.
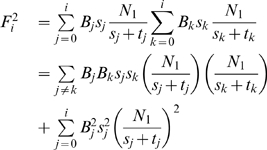



When we take the expected value of 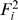 the first term cancels since 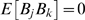 for any 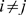. Hence, the variance of 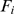 can be computed as:
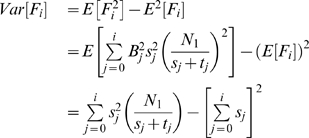



There are many ways to build an estimator from 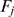
s. However, it is desirable to build an estimator with a small variance as it converges to true solution faster. The following lemma builds the estimator with minimum variance.

#### Lemma 3

*The estimator that has the smallest variance is*

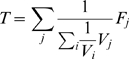



#### Proof

Now, we discuss how we combine the estimators 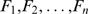 with variances 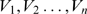 to minimize the overall variance of our estimation. In other words, we want to find the weight parameters 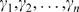 such that 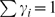 and the variance of the estimator for total number of steady states of Type 1 is minimized. Let us denote this new estimator as 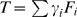. Then,
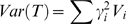



Mathematically, our aim is to minimize 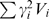 given 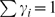. We formulate this problem by using Lagrange Multiplier as follows:
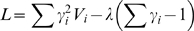



Taking derivative of both sides with respect to each 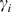, we get the equations:
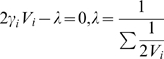



Solving these equations we get the 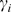 values that minimizes the 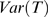 as:
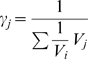



Thus, by using the value of 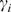
s we find that the estimator with smallest variance is
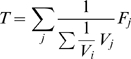



Next, we give the formulations of the estimators for the fractions of each gene and each gene pair being active in steady states. First, we formulate our estimator for the fraction of a gene being active in cyclic steady states. Assume that the number of steady states at the 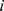
th observation in which the 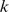 th gene is active is 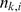. An estimator for the 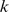 th gene after the 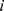
th iteration is then :
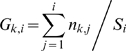
(4)


Let 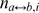 denote the number of steady states in which gene 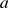 and gene 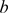 are both active or both inactive after the 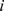
th observation. We calculate the estimator of joint probability of two genes having the same activity level at a steady state as:
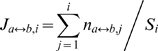
(5)


### Step 4. Stopping Criteria

When our method finishes traversing all Type 1 states (steps 1 to 3), it finds all the steady states. However, in some applications it might be sufficient to find a predetermined percentage of steady states. We develop a statistical criterion to be able to terminate the algorithm quickly after a sufficient portion of the Type 1 states are explored. Our method still guarantees that the desired percentage of the results are found with high confidence. More precisely, when the user supplies a parameter 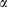 (e.g. 0.9), we compute a confidence 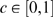, at each iteration such that “*at least* 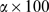 *percent of the steady states are found with probability at least* 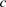
”. This is desirable as the user can terminate the loop when 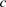 is large enough for the underlying application.

Now, let us describe how the stopping criterion works. Let 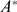 denote the actual number of total Type 1 steady states. If we have known the value of 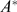 we could have stopped sampling with a confidence value of 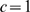 when 
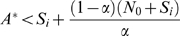
 is satisfied. That is the time when we are sure that 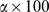 percent of the steady states are already reported. Since we do not know 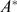 in advance, we use the information gathered from observed portion of states. We compute 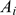 which denotes the minimum number of total steady states of Type 1 that needs to be present for our method to continue traversal.
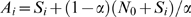
(6)


Trivially, if 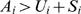 we just stop sampling with 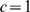 since even if all the unobserved states were to be steady, the reported ones would constitute at least 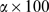 percent of the Type 1 steady states. Otherwise, we calculate the confidence value in 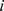
th iteration as the probability that we would have observed at least 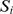 steady states in our observations so far if there were 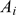 unobserved steady states. Formally, we compute the confidence as:
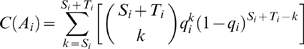
(7)

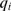
 in Equation 7 represents the percentage of steady states if there were 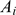 steady states in Type 1 states (i.e. 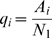). The inner term of the summation represents “The probability of getting exactly 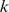 steady states from 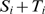 currently observed states if the probability of a state being steady is 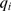
”.

Lemma 4 shows that, the confidence value reported when we stop sampling is never an over estimation.

#### Lemma 4

*The confidence value given in* *Equation 7* *by using* 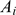 *does not lead to false dismissal.*

#### Proof

Here, we have three cases to consider:

- *Case1 :* (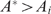)
  Then, 
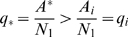
. Since the confidence value is calculated as the area under the right hand side of the probability distribution function (i.e. inverse CDF), 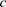 will be larger for a larger value of 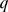. Hence, 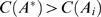. That means whenever we stop sampling the confidence we report is conservative.
- *Case2 :* (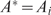)
  Trivially, 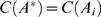 when we terminate the sampling.
- *Case3 :* (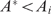)
  This case implies that we overestimated the total number of Type 1 steady states at 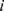
th iteration. Only thing that can happen in such a case is that our method decides to continue traversing when it does not need to. Since the actual number of steady states are less than what we have estimated, when the traversal stops we have already sampled at least as many steady states as needed to guarantee the reported confidence value.
  Corollary 1 follows from Lemma 4.

#### Corollary 1

*Our method guarantees to find all the steady states when the confidence value reaches* 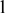 *.*
